# The Effect of Biofeedback Therapy on Anorectal Function After the Reversal of Temporary Stoma When Administered During the Temporary Stoma Period in Rectal Cancer Patients With Sphincter-Saving Surgery: The Interim Report of a Prospective Randomized Controlled Trial

**DOI:** 10.1097/MD.0000000000003611

**Published:** 2016-05-06

**Authors:** Bong-Hyeon Kye, Hyung-Jin Kim, Gun Kim, Ri Na Yoo, Hyeon-Min Cho

**Affiliations:** From the Department of Surgery, St Vincent's Hospital, The Catholic University of Korea, Korea.

## Abstract

We evaluated the effect of biofeedback therapy (BFT) on anorectal function after stoma closure when administered during the interval of temporary stoma after sphincter-preserving surgery for rectal cancer.

Impaired anorectal function is common after lower anterior resections, though no specific treatment options are currently available to prevent this adverse outcome.

Fifty-six patients who underwent neoadjuvant chemoradiation therapy after sphincter-preserving surgery with temporary stoma were randomized into 2 groups: group 1 (received BFT during the temporary stoma period) and group 2 (did not receive BFT). To evaluate anorectal function, anorectal manometry was performed in all patients and subjective symptoms were evaluated using the Cleveland Clinic Incontinence Score. The present study is a report at 6 months after rectal resection.

Forty-seven patients, including 21 in group 1 and 26 in group 2, were evaluated by anorectal manometry. Twelve patients (57.1%) in group 1 and 13 patients (50%) in group 2 were scored above 9 points of Cleveland Clinic Incontinence Score, which is the reference value for fecal incontinence (*P* = 0.770). With time, there was a significant difference (*P* = 0.002) in the change of mean resting pressure according to time sequence between the BFT and control groups.

BFT during the temporary stoma interval had no effect on preventing anorectal dysfunction after temporary stoma reversal at 6 months after rectal resection. However, BFT might be helpful for maintaining resting anal sphincter tone (NCT01661829).

## INTRODUCTION

Impaired anorectal function is common after sphincter-preserving surgery (SPS) for rectal cancer. Currently, with the development of multimodal treatments for rectal cancer, up to 80% of patients with rectal cancer undergo SPS. However, about 60% to 90% of patients who have SPS undergo subsequent changes in bowel habits, with symptoms ranging from daily incontinence to obstructed defecation and constipation, collectively known as anterior resection syndrome (ARS).^1,2^ Historically, depression was significantly more prevalent after abdomino-perineal resection (APR), after which patients had a permanent stoma, than after SPS. Patients with low rectal cancer who are treated by SPS have a superior quality of life (QoL) than those treated by APR.^3^ However, some reports published in the 2000 demonstrated that the QoL for patients after APR was similar to that for patients after SPS. This finding was attributed to the presence of ARS symptoms in patients who received SPS.^4,5^

There are currently no specific treatments for ARS. Management is empirical and symptom-based, using existing therapies for fecal incontinence, fecal urgency, and rectal evacuatory disorder, including loperamide, anal plugs, biofeedback therapy (BFT), rectal irrigation, and neuromodulation with sacral or tibial nerve stimulation.^1,6–9^ Some investigators have reported the favorable effects of pelvic floor rehabilitation on anorectal function. Particularly, BFT has the advantage of providing patients with information about the activity of the pelvic floor muscles by way of a visual display. BFT is considered to be a safe, noninvasive, and inexpensive procedure, with practically no adverse effects.^1,6,7^ However, most studies on ARS have focused on treatments after the occurrence of ARS. Reports on the prevention of ARS, or the appropriate indications and optimal timing of the therapeutic approaches for ARS mentioned above, are rare.

From March 2012, we conducted a prospective randomized controlled study to evaluate the effect of BFT given during the temporary stoma period on the defecation function after stoma closure in patients who underwent SPS with diverting stoma after neoadjuvant chemoradition therapy (nCRT). In the present study, the data were analyzed to evaluate the effect of BFT during temporary stoma on the defecation function at about 6 months after SPS.

## METHODS

This study is registered on clinicaltrials.gov (NCT01661829).

### Ethics

After obtaining review board approval from St Vincent's Hospital, The Catholic University of Korea, CMC Clinical Research Coordination Center (VC12EISI0023), patients were enrolled in the study and their clinical information was prospectively collected.

### Eligibility Criteria

#### Inclusion Criteria

For inclusion in this study, patients must fulfill the following requirements preoperatively^1^: pathologically proven adenocarcinoma^2^; primary tumor located at the rectum up to 12 cm above anal verge (all enrolled patients were confirmed to this item after rigid proctosigmoidoscopy before nCRT)^3^; fecal continence maintained well before nCRT^4^; long-course nCRT (1.8 Gy/day, 5 fractions per week, and a total dose of 50.4 Gy/28 fractions + 2 cycles of concurrent chemotherapy with radiotherapy [5-fluorouracil {5-FU}, 400 mg/m^2^ (i.v.) 1 hour before radiotherapy and leucovorin, 20 mg/m^2^ (i.v.) immediately before each dose of 5-FU on days 1–5 and days 29–33])^5^; temporary stoma during SPS at 6 to 10 weeks after nCRT^6^; adequate organ functions^7^; and written informed consent.

#### Exclusion Criteria

Exclusion criteria are as follows: fecal incontinence before nCRT^2^; previous diverting stoma before SPS due to obstructive lesion, bleeding, fistula, and so on^3^; active infectious disease requiring systemic therapy^4^; and pregnant women.

### Randomization and Sample Size

On conduction of nCRT after SPS with temporary stoma, patients were randomly assigned 1:1 to receive either BFT or no BFT. By using a random-number table with assignment codes concealed in opaque envelopes, half of the patients were randomized to the BFT group and half to the control group. Informed consent was obtained from each participant before random assignment. We used a 2-tailed test with a significance level of 0.05. To meet the sample size requirement, we used a power of 80%. To detect 35% reduction in the incidence of ARS at 1 year after SPS, the estimate of the sample size indicated a total of 56 patients would be required: 28 patients randomized to BFT group and 28 to the control group.

### Primary End Point

Our primary goal was to identify the difference in the incidence of defecation dysfunction and the recovery time needed at 1 year after SPS with temporary stoma between 2 groups. The present study is an interim analysis at 6 months after SPS with temporary stoma.

### Study Design

From March 2012 to February 2014, a total of 56 patients who underwent nCRT after SPS with temporary stoma were enrolled in our study. BFT (n = 28) was performed 1 or 2 times a week during the temporary stoma interval. Conservative self-rehabilitations, such as Kegel exercises, were advised to patients randomized to the control group (n = 28), and also the BFT group. To evaluate the anorectal function, we performed anorectal manometry, transanal ultrasound, and Cleveland Clinic Incontinence Score (CCIS) at the following time points: before nCRT (period 1), after nCRT (period 2), before the reversal of temporary stoma (period 3), 6 months after SPS with temporary stoma (period 4), and 12 months after SPS with temporary stoma (period 5). Patients were randomly assigned to 1 of the 2 groups just before first adjuvant chemotherapy after SPS with temporary stoma. We evaluated subjective defecation symptoms by CCIS, mean number of defecation during 1 day, severity of incontinence (none, urgency to evacuate, soiling, accidents), and the use of antidiarrheal drugs, at each period. We also estimated the treatment response with objective parameters using anorectal manometry. The “degree of change” was regarded as the rate of change of the manometric data based on data from period 1 (manometric data in each period/manometric data in period 1). Additionally, the response was measured by the mean value of the individual “degree of change” at each period (“measure of response”). In present study, we used the “degree of change” and the “measure of response” as the main comparative parameters between the BFT and control groups. In addition, we compared the “measure of response” according to the initial tumor location from anal verge and method of colorectal or coloanal anastomosis. The patients were classified into 2 groups based on the location of the tumor—one with the lower margin of remaining more than 5 cm and the other with the lower margin of less than 5 cm. The distance of the lower margin was measured by rigid proctosigmoidoscopy. For the patients with the lower margin remaining less than 5 cm, the anal canal was included in the field of the additional boost given to the peritumoral region. For the patients with the tumor located above 5 cm from the anal verge, the anal canal and perineum were hindered from an additional boost. Colorectal anastomosis (CRA) was performed using the double stapling technique through an intra-abdominal approach. Coloanal anastomosis (CAA) was performed using the hand-sewn technique through a perianal approach. Currently, we have completed follow-up for data collection to period 5 and this interim analysis was performed with data collected up to period 4 (6 months after SPS).

### Statistical Analyses

Continuous variables were compared using the Student *t* test and one-way analysis of variance (ANOVA), and expressed as mean ± SD. Categorical variables were analyzed with the chi-square test. The “degree of change” and “measure of response” according to various factors at each period were compared with the generalized linear models. Significance was defined as a *P* value ≤0.05. All statistical analyses were performed using the Statistical Package of the Social Sciences (SPSS) version 15.0 for Windows (SPSS, Inc., Chicago, IL).

### Role of the Funding Source

This study was funded by The Catholic Medical Center Research Foundation in the 2013 program year. The study sponsor had no involvement in the trial design, collection, analysis, or interpretation of data, or the writing of the report. The corresponding author had full access to all data and had the final responsibility for the decision to submit the report for publication.

## RESULTS

**FIGURE 1:**
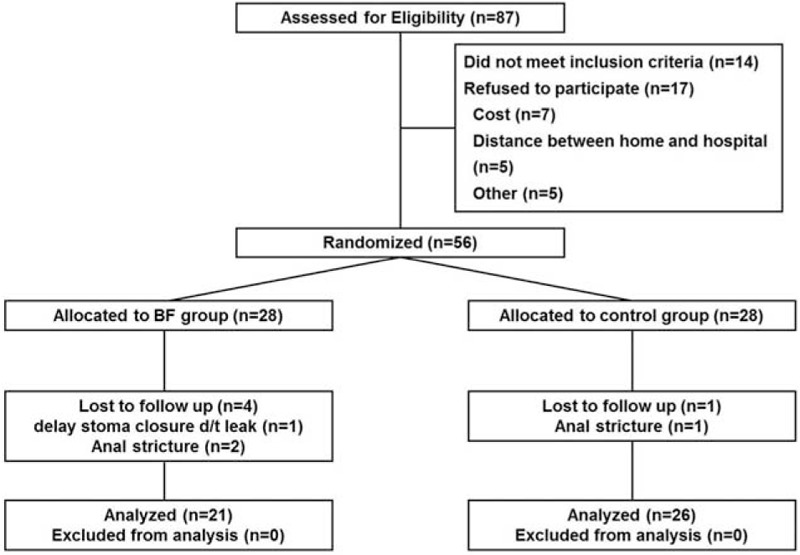
Flowchart of the inclusion process.

Figure 1 shows the flow of participants through the recruitment and randomization phase of the study. A total of 87 patients were screened for eligibility. Of these, 14 patients did not meet the inclusion criteria, 17 patients declined to participate in this study, 7 patients declined physiological tests at each period due to economic problems, 5 patients lived at a long distance from our hospital, and 5 patients refused enrollment in this study for unknown causes. In all, 56 patients, for whom baseline measurements were taken before randomization, consented and were subsequently randomized to an intervention condition. In the BFT group, 4 patients were lost to follow-up, 1 who received delayed stoma closure underwent an anastomosis leak after lower anterior resection, and 2 could not receive physiological testing due to postoperative anal strictures. In the control group, 1 patient was lost to follow-up and 1 had anal stricture. Ultimately, 21 patients in the BFT group and 26 patients in the control group received physiological testing on the study schedule at period 4.

Demographic and baseline characteristics of the study sample are presented in Table 1. BFT and control participants did not differ significantly (*P* > 0.05) on any demographic or characteristic at baseline.

**TABLE 1:**
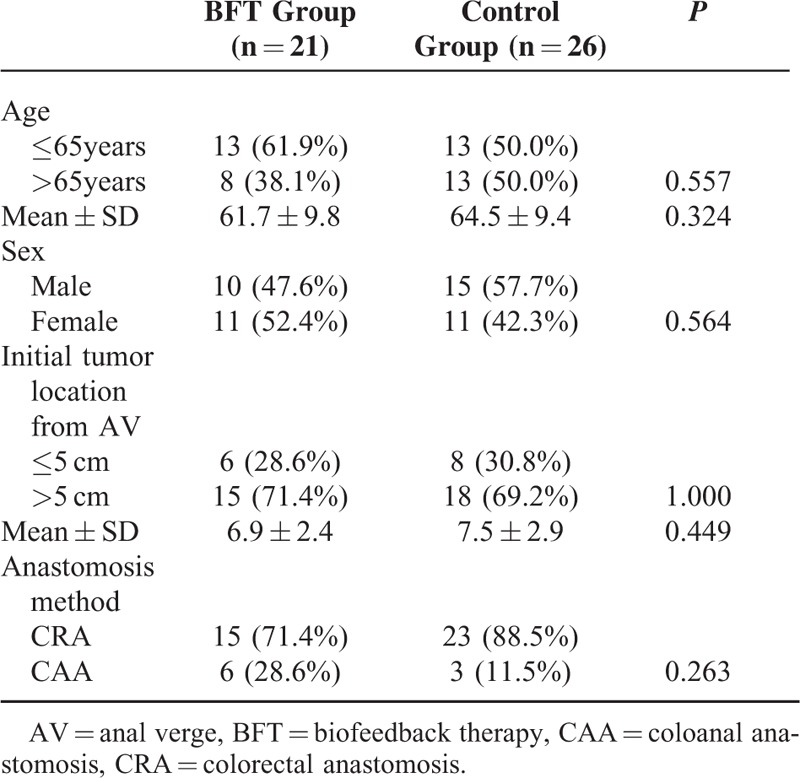
Patient Demographics and Clinical Characteristics

The “measure of response” according to each manometric parameter at period 4 is presented in Table 2. There were no significant differences in the “measure of response” in any manometric parameters at period 4 according to treatment options (BFT group vs control group), initial tumor location (≤5 vs >5 cm), or anastomosis method (CRA vs CAA).

**TABLE 2:**
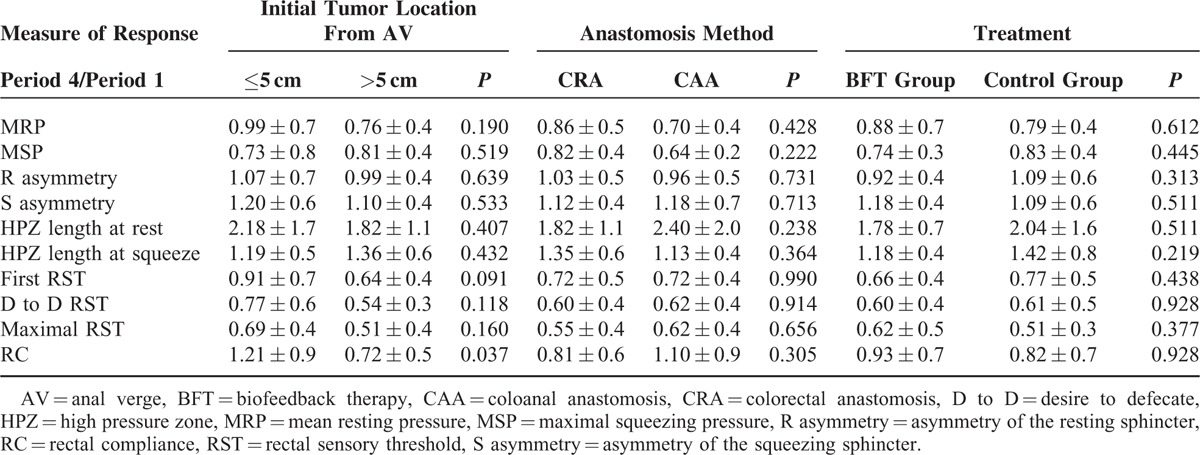
Anorectal Function Reflected by the “Degree of Change” at Period 4

Table 3 shows the defecation function of our patients at period 4. CCIS, severity of incontinence, use of antidiarrheal drugs, and the number of daily defecations were not significantly different based on the treatment options, initial tumor location, or anastomosis method. However, patients who underwent CAA more frequently complained of incontinence for solid stool (*P* = 0.046) at period 4. Most patients with fecal or gas incontinence at period 4 had suffered from an “urgency to evacuate” stool or gas.

**TABLE 3:**
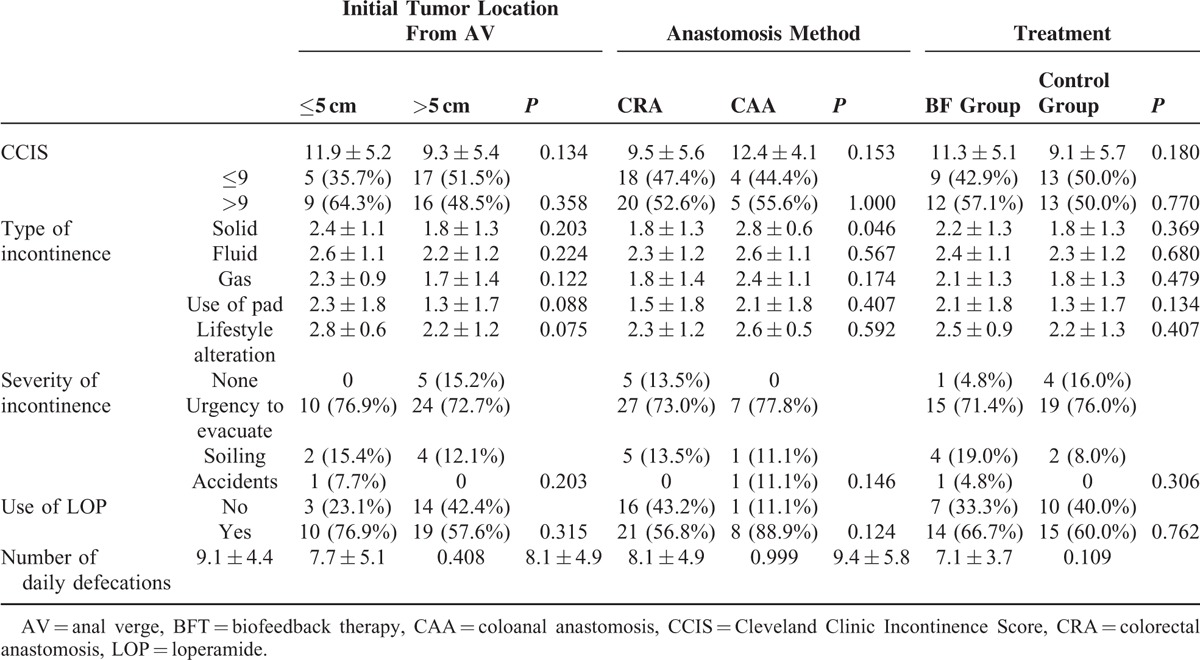
Analysis of Defecation Function at Period 4

**FIGURE 2:**
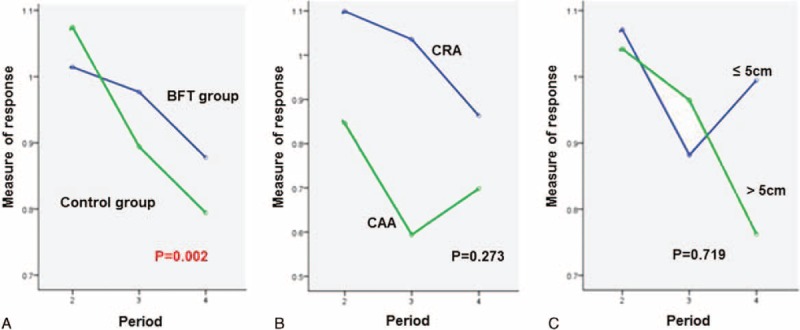
Changes in the “measure of response” for mean resting pressure (MRP) with time. There was significant difference in the change in the “measure of response” for MRP between the BFT and control groups (*P* = 0.002). The change in the “measure of response” for MRP is shown according to (A) treatment options, (B) anastomosis method, or (C) initial tumor location. BFT = biofeedback therapy.

**FIGURE 3:**
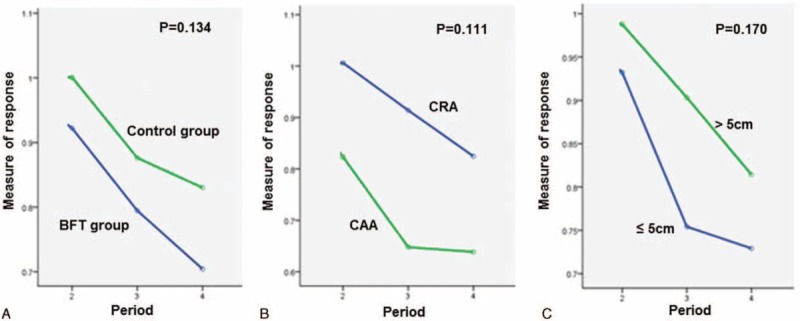
Changes in the “measure of response” for maximal squeezing pressure (MSP) with time. There was no significant difference in the change in the “measure of response” for MSP according to (A) treatment options, (B) anastomosis method, or (C) initial tumor location.

**FIGURE 4:**
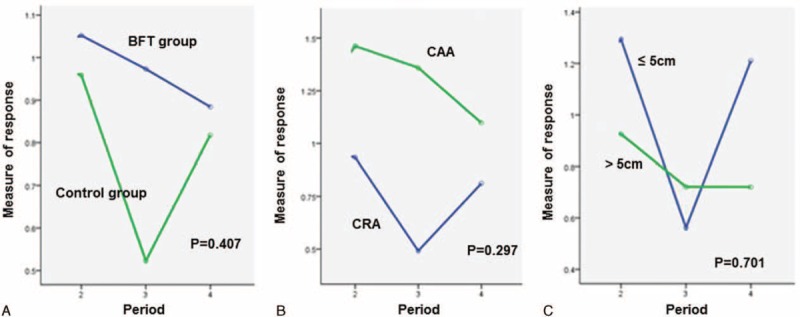
Changes in the “measure of response” for rectal compliance (RC), with time. There was no significant difference in the change in the “measure of response” for RC according to (A) treatment options, (B) anastomosis method, or (C) initial tumor location.

Figure 2, Figure 3, and Figure 4 present the changes in the “measure of response” for the mean resting pressure (MRP), maximal squeeze pressure (MSP), and rectal compliance (RC), respectively, across the course of time. There was significant difference in the change in the “measure of response” for MRP according to time sequence between the BFT and control groups (*P* = 0.002). However, there were no significant differences in the change in the “measure of response” for MSP and RC according to time sequence between the 2 treatment groups. In addition, there were no significant differences in the change in the “measure of response” for MRP, MSP, and RC according to the anastomosis method and the initial tumor location from the anal verge across the course of time.

## DISCUSSION

Recently, with the advancements in surgical techniques, SPS for mid to low rectal cancer is widely adopted by colorectal surgeons.^1,2^ Additionally, nCRT followed by SPS is a popular treatment option for mid to low advanced rectal cancer.^10,11^ By avoiding permanent stoma, SPS provides an opportunity to prevent any changes in the patients’ body structure, which is crucial for the physical and emotional well being of individuals with rectal cancer. However, about 60% to 90% of patients who undergo SPS suffer from subsequent changes in bowel habits, known as ARS.^1,2^

Several studies have been performed to prevent or treat ARS. Firstly, several surgeons have tried to change the neorectal configuration with diverse anastomotic techniques, including colonic J-pouch or coloplasty.^12^ Though these techniques were tried to improve rectal compliance, there were no obvious long-term benefits of any particular technique.^12–14^ Secondly, empirical and symptom-based treatment with loperamide has frequently been used in the clinic. Loperamide acts directly on the intestine to inhibit peristalsis, lengthens the small intestinal and mouth to cecum transit time, increases the sphincter tone and resting pressure, and reduces urgency, stool volume, and the frequency of bowel movements. It also reduces the sensitivity of the rectoanal inhibitory reflex (RAIR) and increases rectal perception in healthy subjects.^3,14^ BFT is an established treatment option for constipation and fecal incontinence. With BFT, the patient gets information about activity of the pelvic floor muscles by way of a visual display. One systematic review demonstrated the utility of pelvic floor rehabilitation for improving the functional outcome after a lower anterior resection. A majority of the studies included in this review showed an improvement in continence, stool frequency, and overall quality of life.^15^ Lastly, neuromodulation has recently been applied in patients with ARS. Sacral nerve stimulation (SNS) in adults with fecal incontinence that is not responsive to medical therapy results in an improvement of over 50% in symptoms in approximately 80% of patients.^8,16^ One systematic review of SNS for ARS demonstrated that, in light of the apparent symptomatic benefit, it is clearly worth attempting SNS in patients with ARS not responding to medical treatment, particularly owing to the low risk of complications.^16^

However, despite the high incidence of ARS after SPS, most treatments of ARS, except the anastomotic technique, have been applied after the occurrence of ARS. Anastomotic height or the extent of operation, postoperative chemotherapy, radiation therapy, and temporary stoma are risk factors for ARS.^17^ All patients enrolled in this study received nCRT, temporary stoma, and postoperative adjuvant chemotherapy. Therefore, all patients in our study were at a high risk for developing ARS. Patients who undergo SPS with temporary stoma have an anal-resting phase for about 10 weeks before the closure of temporary stoma. However, during this interval, patients do not receive any special support for preventing or minimizing ARS. The present study was designed to assess whether BFT represents a promising intervention during this interval to prevent or minimize ARS.

Generally, most surgeons may recommend pelvic muscle rehabilitation, such as Kegel exercises, to their patients during the anal resting phase with temporary stoma. The aim of Kegel exercises is to improve muscle tone by strengthening the pubococcygeus muscles of the pelvic floor. It is now known that with Kegel exercises, the components of the levator ani muscles contract and relax as one muscle. This type of exercise may be beneficial in cases of fecal incontinence and pelvic organ prolapse.^18^ However, the correct execution of these exercises is not checked by medical staff, making it difficult to determine whether the training was ineffective owing to inherent inefficiency, or because it was incorrectly performed.^19,20^ On the contrary, BFT can give the patient information about the activity of the pelvic floor muscles by way of a visual display. Hence, BFT can give the patients information about the improvement in their pelvic floor muscle strength, which can also be monitored by medical staff.^15^ This is a key difference between BFT and Kegel exercises. BFT is an established treatment for constipation and fecal incontinence. One nonrandomized retrospective study assessing the effectiveness of BFT for ARS showed a significant improvement in fecal incontinence scores and bowel frequency. BFT is noninvasive, inexpensive, with minimal adverse effects.^1^ BFT in addition to Kegel exercises may be a suitable and safe option to apply during the anal resting phase with temporary stoma.

In our study, about half the enrolled patients had high CCIS (more than 9 points) and about 70% of patients complained of the “urgency to evacuate” at period 4 (Table 3). These findings were not different according to treatment options, initial tumor location, or the anastomosis method. We postulate that the “urgency to evacuate” after rectal resection may be related to neorectal capacity and compliance. Although there is no direct evidence that anorectal manometry reflects a real anorectal defecatory function, 1 study suggested that anorectal manometry demonstrated excellent sensitivity, a moderate specificity, and convincing accuracy.^21^ Therefore, the discriminatory power of anorectal manometry in the evaluation of fecal incontinence patients is sufficiently high to justify its clinical use. With this in mind, anorectal manometry was applied in the present study to interpret our findings. We found that lower tumor location was significantly related to greater rectal compliance (*P* = 0.037). For all our patients, intestinal conduits were made by straight end-to-end anastomoses after rectal resection. Generally, we have more frequently performed hand-sewn CAA for tumors located within 5 cm from the anal verge, and double-stapled CRA for tumors located above 5 cm from the anal verge. The neorectum, made by staple anastomosis, might be less distensible than that made by hand-sewn anasotmosis. In addition, though a certain extent of irradiated rectum might remain after rectal resection for tumors located above 5 cm from anal verge, the irradiated rectum seldom remained after rectal resection in tumors located within 5 cm from the anal verge. The irradiated rectum may be a factor contributing to lesser distensibility of the neorectum. Bregendah et al^22^ demonstrated that leaving an irradiated remnant rectum as the distal part of the anastomosis may be an important factor in the further impairment of functional outcome, possibly due to decreased neorectal sensitivity of the remnant rectum related to visceral afferent nerve dysfunction and fibrosis. Bondeven et al^23^ reported that although both the length of remnant rectum and nCRT had a major impact on the severity of bowel dysfunction after restorative rectal cancer surgery, no functional benefit from an irradiated rectal remnant was observed. Our results support these findings. We hypothesize that the anastomosis method and remnant irradiated rectum might explain the association between the lower tumor location and greater rectal compliance.

Another factor related to the “urgency to defecate” may be rectal or anal hypersensitivity. In our study, the rectal sensory threshold (RST) was indicative of rectal hypersensitivity and MRP was indicative of anal hypersensitivity. Although rectoanal coordination, reflected by the RAIR, is more important for rectoanal hypersensitivity,^24^ several patients had not yet recovered this coordination at the time of period 4.^25,26^ Accordingly, RAIR was replaced with MRP at that time point for the evaluation of anal hypersensitivity. The “measure of response” for MRP was not significantly different between the BFT and control groups (*P* = 0.612) at period 4 (Table 3). However, the change in the “measure of response” for MRP according to time sequence between the BFT and control groups were different (*P* = 0.002; Figure 2), which suggested that patients who were administered BFT during the temporary stoma phase maintained their MRP better than those who performed Kegel exercises only. In other words, BFT was effective for the prevention or minimization of the deterioration of anal hypersensitivity. MRP reflects the involuntary internal anal sphincter status. In addition, the internal anal sphincter plays a part in the RAIR, which implies rectoanal coordination in addition to rectal compliance. Consequently, it may be essential to maintain the internal anal sphincter function in conditions where rectal compliance would be decreased after rectal resection. A French study for SNS in 200 fecal incontinence patients demonstrated that the stool consistency and low stimulation intensity were predictive factors for a successful outcome of SNS.^27^ According to the analysis of the manometric data in this study, a higher MRP before SNS was related to success at the 6-month follow-up after permanent implantation.^27^ Taken together, BFT during temporary stoma may be a potentially useful treatment strategy for ARS and for eliciting improved responses for future SNS. However, because the present study is a report at 6 months after rectal resection, the effect of BFT on anorectal function at 1 or 2 years after rectal resection remains to be evaluated.

The limitations of our study are that a small number of patients were included, and sufficiently detailed instructions for Kegel exercises were not provided. In addition, at period 4, antidiarrheal drugs were prescribed without any criterion. However, there are not any reports for improving ARS with treatment modality during temporary stoma after rectal resection. Even though BFT during the temporary stoma phase failed to improve the symptomatic scores, we found that BFT during this interval might be effective for the maintenance of anal hypersensitivity. Moreover, BFT during temporary stoma may provide better conditions for the application of additional treatments, including SNS. We are presently analyzing the effect of BFT on the defecation function at period 5. Future data will further elucidate the relationship between the results obtained by period 4 and defecation function at period 5.
